# Flow Cytometry: Advances, Challenges and Trends

**DOI:** 10.1002/bies.70091

**Published:** 2025-11-27

**Authors:** J. Paul Robinson, Grzegorz B. Gmyrek, Bartek Rajwa

**Affiliations:** ^1^ Basic Medical Sciences Purdue University West Lafayette Indiana USA; ^2^ Weldon School of Biomedical Engineering Purdue University West Lafayette Indiana USA; ^3^ Miftek Corporation West Lafayette Indiana USA; ^4^ Bindley Bioscience Center Purdue University West Lafayette Indiana USA

**Keywords:** cell sorting, fluorescence, light scatter, monoclonal antibody, photon detection, polychromatic flow cytometry, spectral flow cytometry

## Abstract

Flow cytometry is a versatile analytical technology for measuring physical and molecular characteristics of individual cells or particles in suspension. The technology has had its greatest impact in immunology, enabling the identification and quantification of rare cell populations within complex mixtures, but applications span diverse biological systems including hematopoietic cells, microorganisms, cultured cells, plant cells, gametes, and disaggregated tissues. Target molecules are typically identified using fluorophore‐conjugated antibodies, though alternative labeling strategies exist. A key advantage of flow cytometry is the ability to physically isolate cells of interest for downstream applications such as culture, genomic analysis, or functional studies. The field has undergone substantial evolution from conventional filter‐based polychromatic systems to spectral cytometry platforms that capture full emission spectra, enabling higher‐parameter analyses and more flexible panel design. This review examines current capabilities and limitations of flow cytometry technology, with emphasis on recent advances in spectral detection, quantitative standardization, and computational analysis. We discuss remaining technical challenges and explore emerging opportunities for innovation in excitation systems, detector technology, and integration with artificial intelligence‐based analysis platforms. Addressing these challenges will be essential for cytometry to continue driving biological discovery and clinical applications in the coming decades.

## Graphical abstract

Recent advances in flow cytometry span hardware, data processing, and AI‐driven analytics. Emerging modalitiesincluding spectral, imaging, mass, and nano/EV cytometryenable higher‐parameter and small‐particle detection. Integrated machine learning drives a shift from manual gating toward automated multi‐omics workflows. Future trends in flow cytometry include real‐time functional profiling, standardized cloud‐based data pipelines, and single‐photon counting to deliver deeper cellular insights.

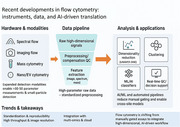

**Abbreviations**
PC: polychromatic cytometry
SC: spectral cytometry
OMIPs: optimized multicolor immunofluorescence panels
SSM: spillover spreading matrix
PMT: photomultiplier
APD: avalanche photodiode detector
SQI: spread quantification index
CL: cell ontology
COG: Children's Oncology Group
FCS: Flow Cytometry Standard
tSNE: t‐distributed stochastic neighbor embedding
UMAP: uniform manifold approximation projection
CE‐IVD: in vitro diagnostic
IVD: in vitro diagnostic
PI: propidium iodide
DAPI: 4′,6‐diamidino‐2‐phenylindole
DJF: Dean‐Jett‐Fox
WP: Watson Pragmatic
PSM: probability state modeling
EVs: extracellular vesicles
LOD: limits of detection
TO: thiazole orange
WGA: wheat‐germ agglutinin
CTC: 5‐cyano‐2,3‐ditolyl‐tetrazolium chloride
FACS: fluorescence‐activated cell sorting
IACS: image‐activated sorters include
CW: continuous wave
ISAC: International Society for the Advancement of Cytometry
RUO: research use only
CRO: contract research organizations

## 1 Introduction

Flow cytometry has repeatedly solved the same problem: how to measure cellular heterogeneity at a scale and resolution that matches its biological complexity. The technique has been employed in biological research for roughly six decades, originating from efforts to determine whether two distinct red blood cell populations were indeed different. This challenge led Mack Fulwyler to develop a novel instrument based on the principle of impedance [1]. Soon thereafter, fluorescence‐based detection proved superior, as demonstrated by Göhde [2] and Herzenberg [3], who also introduced a sorting capability to fluorescence measurements. From these early developments, flow cytometry evolved into a powerful tool for single‐cell fluorescence analysis. Continued improvements in instrumentation, along with the development of new fluorochromes and antibodies, enabled the simultaneous detection of numerous fluorescent signals. The advent of monoclonal antibodies [4] and the implementation of multiple detectors further expanded the capacity of flow cytometry to identify and separate a wide variety of cell populations. Today, the field confronts this challenge again, as conventional approaches have reached their practical limits.

## 2 Fundamentals of Flow Cytometry

**FIGURE 1:**
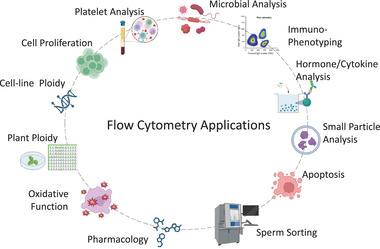
Shown are a variety of applications that can be used with flow cytometry. Not all applications can be achieved on every instrument, but there are a large variety of instruments that have different features such as lasers, detectors, fluidic devices, and so forth, that might be necessary for one or other applications.

Most flow cytometers comprise four fundamental components: a fluidics unit, an excitation system, an emission system, and core electronics, as well as the analysis and processing software. Although the majority of flow cytometers function similarly, there are significant distinctions in the operation of their detection and analysis components. Systems consist of analyzers and sorters, with the sorter having the capacity to physically separate cells at very high speed and under sterile conditions. Some current instruments are capable of producing relatively low‐resolution images that can be used to detect cells. Figure 1 highlights the diverse applications of flow cytometry, which are examined in greater detail in the subsequent sections.

When it comes to analyzers, there are two main approaches: polychromatic cytometry (PC), which assigns each fluorochrome to a specific detector, and spectral, which considers the spectrum as the measurable feature. Both will be discussed; while polychromatic instruments are currently the most popular among users, the trend is toward spectral instruments. It is highly likely that within 5 years, the majority of new instruments will be spectral, causing polychromatic instruments to disappear.

Polychromatic cytometry has been the predominant technology for approximately 50 years and constitutes the majority of installed instruments. From the earliest days of cytometry, it was recognized that increasing the number of measurable fluorochromes was highly desirable. The first instruments collected signal from just a single fluorochrome [5], then 2 [6], 5 [7], 8 [8], and 11 colors [9]. By this point, it was clear that immunophenotyping was the driving force behind the field, and the goal was to identify as many subsets of markers as possible. By 2004, 17‐color studies had been conducted, revealing the limitations of PC cytometry due to a lack of space in the visible spectrum for additional dyes [10]. The apparent solution was to increase the number of lasers and the number of detectors in combination with new reagents, particularly the Brilliant Violet dyes developed by Sirigen [11].

The complexity of handling compensation (a cytometry vernacular for signal demultiplexing), a prerequisite for the successful expansion of the assay complexity, rose in tandem with the number of fluorochromes. Compensation, originally defined in detail by Bagwell and Adams [12], involved adjusting each detector setting to “compensate” for signals originating from other spectrally overlapping fluorochromes. As the complexity of the assay increases, so too does the difficulty of establishing and implementing a compensation process.

Because of the difficulty in creating narrow bands within the visible spectrum, the limited number of lasers available, and the inevitable spectral overlap, PC cytometry was fundamentally limited to fewer than 30 fluorescent probes at any given time.

Spectral cytometry was originally developed by our group at Purdue in the early 2000s and first published in 2004 [13, 14]. However, the technology was not commercialized until 2015 [15], when it quickly became the preferred approach as assay complexity increased to 40 colors [16] and 50 colors [17], effectively doubling the potential number of fluorophores in polychromatic cytometry.

**FIGURE 2:**
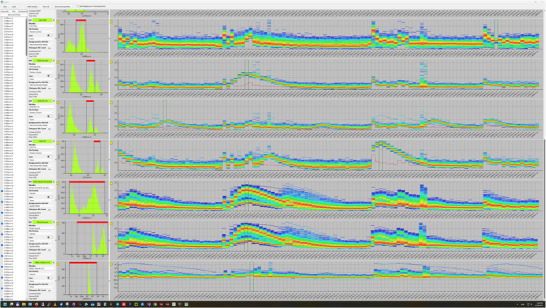
Spectral flow cytometry allows for the full spectral analysis of cellular systems in such a way to extend the number of fluors that can be used simultaneously.

Spectral cytometry offers several fundamental advantages over conventional techniques. Because its detection system does not rely on adjustable optical filters, automation is inherently simpler. This stability enhances reproducibility, as the spectral unmixing process can be performed consistently and independently of the operator. Moreover, spectral cytometry has demonstrated a markedly greater capacity to analyze a broader range of fluorochromes and, consequently, a larger number of cellular subsets than traditional PC. While immunologists often recognize this expanded analytical range as a major benefit, additional implications are sometimes overlooked. For instance, spectral cytometry's ability to demultiplex highly overlapping emission spectra enables simultaneous analysis of molecular, enzymatic, nucleic acid, and structural markers alongside conventional immunophenotypic markers. This concept is illustrated in Figure 2, which depicts the concurrent spectral analysis of various fluorescent dyes bound to cells.

## 3 Current Applications of Flow Cytometry

### 3.1 Immunophenotyping

Immunophenotyping is widely acknowledged as the primary application of flow cytometry, making it one of the most utilized techniques for identifying specific cell populations within a sample. This is accomplished by analyzing the expression of multiple surface and intracellular markers using a wide range of fluorochrome‐conjugated monoclonal antibodies to discover new biomarkers associated with disease onset/progression or to monitor the efficacy of treatment [18, 19]. Among the key factors that impact immunophenotyping, in terms of preparation and data interpretation, are staining panel design [20], cell annotation [21], and the versatile use of analytical tools for cell data analysis [22].

#### 3.1.1 Panel Design

Advances in immunophenotyping, particularly performed using spectral flow cytometry, necessitate a careful approach to staining panel design in order to improve accuracy in profiling immune cells while also managing the complexity of the assay [23]. This is especially significant given that spectral flow cytometer analyzers with five lasers have 51–144 detectors and can measure 15–50 signals at once [17]. However, as Howard Shapiro stated, “there is no magic in staining panel design,” following specific guidelines greatly increases the likelihood of obtaining biological information free of artifacts.

Successful panel design is a multifaceted process that involves several key steps: understanding the biological context, configuring the instrument, selecting antigens and clones, and matching antigen expression and density (including co‐expression of markers) with appropriate fluorochrome brightness. These steps culminate in a theoretical review of the panel. Peer‐reviewed resources such as optimized multicolor immunofluorescence panels (OMIPs) can be instrumental in this initial evaluation, as these panels have been rigorously validated for assessing immune‐cell populations across various species [24, 25, 26, 27].

It is important to note that some OMIPs were designed for specific instruments, which may limit the optimization of staining panels across different flow cytometry platforms. Because fluorochrome brightness indices vary between instruments, adjustments may be required when selecting fluorochromes. Given the vast availability of over 4 million commercially available antibodies and more than 1000 fluorochromes and dyes, as noted on antibodypedia.com and Fluorofinder, determining the appropriate combination of fluorochromes can be both time‐consuming and difficult [27, 28].

As a practical solution, automated panel design tools such as EasyPanel (Dotmatics), IntelliPanel (FluoroFinder), and manufacturer‐provided software like Cytek Cloud offer valuable starting points for efficient panel construction. EasyPanel, for example, automatically matches highly and weakly expressed antigens with appropriately bright or dim fluorochromes based on the user's selected instrument. Within seconds, the software cross‐references selected fluorochrome combinations against commercial databases to assess reagent availability and vendor options, thereby helping users control purchasing costs.

This automation represents a significant improvement, as the design of high‐parameter staining panels has traditionally been both time‐consuming and expensive when performed manually. In addition to theoretical evaluations and performance metrics, these tools can identify the most suitable fluorochrome—antibody combinations for a given instrument configuration. However, it is important to emphasize that automated panel design does not eliminate the need for empirical antibody titration, which remains essential for optimizing signal resolution and overall panel performance [29, 30, 31].

The objective of antibody titration, in addition to identifying the optimal concentration for achieving the best staining resolution, is to help minimize the costs associated with high‐parameter flow‐cytometry panels. Research has demonstrated that extending the incubation time to 16–20 h yields improved resolution and reduced antibody usage compared to the typical 15–30 min of cell staining [32].

The so‐called spreading error (SE) is a major obstacle in high‐parameter flow‐cytometry panel design. It arises when an unmixing algorithm cannot perfectly demultiplex overlapping emission spectra, and it is amplified by photon‐counting noise, whose variance scales with the detected signal [33]. Because cytometric data are typically *super‐Poissonian* (i.e., the variance grows faster than the mean), this noise inflation occurs regardless of detector technology (PMT, APD, or silicon detectors), further exacerbating SE. Using an identical fluorochrome panel on a *spectral* cytometer usually yields sharper population resolution and lower SE than on a conventional (filter‐based) instrument. The improvement is due to two factors: (i) spectral systems use an over‐determined mixing matrix (more detectors than fluorochromes), allowing them to apply noise‐aware, empirically calibrated unmixing models; and (ii) conventional compensation, which assumes a single dedicated channel for each dye, is inherently less efficient [34]. Finally, SE is additive: each additional, spectrally overlapping dye co‐expressed on the same cell increases cumulative variance in downstream channels [35].

Two metrics are routinely used to judge panel performance in flow cytometry. The first is the cosine similarity between the emission spectra of the employed dyes, also known as the *similarity index*, which ranges from 0 (no overlap) to 1 (complete overlap). The second is the condition number of the mixing matrix, often referred to as the *complexity index*, even though it conveys numerical stability rather than biological or numerical complexity. While cosine similarity describes pairwise spectral overlap, it should not be treated as an estimator of spreading error: a low cosine similarity does not guarantee the absence of SE once these fluorochromes are embedded in a larger, multi‐color panel [17]. The condition number assesses the eigenvalues of the mixing matrix and identifies panels that may contain problematic fluorochrome combinations, although it does not specify which pairs may contribute to spreading.

Several empirical surrogates have been proposed to quantify uncertainty in unmixing/compensation. The spillover spreading matrix (SSM), which is the earliest and still most widely used, captures signal spread regardless of the amount of fluorochrome applied, but its values depend strongly on detector sensitivity. Consequently, SSM scores cannot be compared directly across instruments with different dynamic ranges or detector types [33, 35]. The spread quantification index (SQI) overcomes this drawback by normalizing for fluorochrome intensity; as a result, SQI is largely invariant to detector architecture and range [33, 36, 37]

Cytek Biosciences has further introduced a resolution impact matrix, which employs the stain index to (i) summarize the intrinsic resolution of each fluorochrome and (ii) reveal how that fluorochrome diminishes the resolution of others in dual‐marker populations [31].

To move beyond ad hoc heuristics, investigators have proposed a more statistically grounded metric for predicting SE. The strategy utilizes the Variance Inflation Factor (VIF) matrix for the linear unmixing model; by computing the square root of each VIF matrix element (taken in absolute value), one obtains the indication of the standard deviation that any fluorochrome (or fluorochrome pair) can impose on the measurement. This approximate “noise floor” reveals how panel architecture influences variance across all channels, a capability that proves invaluable for complex (>35‐parameter) designs. Indeed, in a simulated 50‐color panel, several dyes lost roughly five‐fold resolution in the negative gate [17]. BD packages this analysis into a *spectral hotspot matrix*, so named because cells with large values (“hotspots”) flag risky fluorochrome combinations. The tool is hosted on the BD Research Cloud and tuned for both BD FACSDiscover S8 and Cytek Aurora analyzers [38].

Last, an area that requires further investigation is the performance of a staining panel optimized for one spectral instrument when applied to another spectral flow cytometer. Cross‐platform portability of spectral panels remains understudied. Although identical laser/detector layouts should, in principle, yield comparable performance, practical differences in brightness indices, SSM values, autofluorescence extraction, hardware settings, and algorithm choice often intervene. A potential solution is to design a foundational staining panel focused on basic markers, allowing for future enhancements. Although no human data are currently available on this topic, studies in mice suggest that this approach could yield promising results [39].

#### 3.1.2 Cell Annotations

Consistent cell‐type annotation is essential for comparing flow cytometry data across experiments, laboratories, and time points, necessitating standardized terminology. The Cell Ontology (CL) provides a consensus‐driven, hierarchical controlled vocabulary that enables researchers to annotate cell populations consistently and unambiguously. Originally established in 2005 [40], CL is continuously maintained and expanded by a community of domain experts, encompassing cell types across diverse tissues and organisms. As of 2025, the ontology contains over 2400 terms, including more than 500 immune cell types with precisely defined relationships and properties [41]. This structured vocabulary facilitates computational integration and comparison of flow cytometry datasets by providing standardized identifiers for cell populations.

Several computational tools leverage CL to automate cell‐type annotation in flow cytometry. The flowCL package implements automated labeling of flow cytometry populations based on marker expression profiles mapped to CL definitions [42]. Machine learning approaches such as OnClass extend automated annotation capabilities by enabling cell‐type prediction across datasets, including identification of rare populations or cell types underrepresented in training data through hierarchical classification strategies [43]. Integration of ontology‐based annotation with AI‐driven analysis pipelines represents a key step toward reproducible, standardized flow cytometry workflows that align with FAIR data.

Despite its advantages, several challenges related to CL in flow cytometry have been identified, including subjectivity in gating and technical difficulties associated with assay qualification and validation [44, 45]. This may lead to variability in flow cytometry measurements across different laboratories and experiments. Several attempts have been made to address standardization in flow cytometry measurements, including initiatives by EuroFlow, ERIC, the Children's Oncology Group (COG), the AIEOP‐BFM group, and the Human Immunology Project. For instance, in 2012, the Human Immunology Project proposed eight‐color antibody staining panels for T cells, Treg, Th1, Th2, Th17, B cells, NK cells, DCs, and monocytes [21]. EuroFlow collected over 6000 anonymized FCS files acquired using conventional instruments to establish an automated system for cell phenotype comparisons, which is implemented in Infinicyt software (Cytognos/BD Biosciences) [45, 46].

Recently, the SOULCAP (Standardized Ontology Unique Labeling for Cytometry Annotation of Populations) initiative has been established to address the critical challenge of inconsistent immune cell‐population labeling in cytometry data. The primary objective of SOULCAP is to develop a unified nomenclature for immune‐cell populations and ensure the widespread adoption of this standard throughout the scientific community through collaboration among academic institutions, pharma and biotech, reagent manufacturers, and software developers [47].

#### 3.1.3 Immunophenotyping Data Analysis

When conventional flow cytometers were equipped with only a single laser and supported three‐color staining assays, manual data analysis was relatively straightforward. The equation for the number of bi‐axial plots (*N* × (*N* − 1))/2, where *N* represents the number of channels or colors, shows that a three‐color assay has three possible combinations. Over time, as cytometry advanced to 10‐color staining, the number of possible combinations increased to 45. The introduction of spectral flow cytometers, which allow the simultaneous evaluation of 45 markers, has expanded this to 990 possible combinations. Consequently, the use of panels with more than 10 colors raises questions about the practicality of traditional manual gating for data analysis and necessitates a shift toward more robust analytical tools (unsupervised or supervised) to effectively manage the increasing complexity of the data [44, 48].

Although numerous new computational approaches for biological data analysis are published each year, only a few achieve widespread adoption for routine use. Examples include dimensionality‐reduction tools such as Uniform Manifold Approximation and Projection (UMAP) and t‐distributed Stochastic Neighbor Embedding (t‐SNE), as well as clustering algorithms like FlowSOM, PhenoGraph, and ClusterExplorer [35].

While these tools are effective for data visualization and inspection, it is crucial to take precautions to ensure that their implementation yields biologically meaningful results. A consideration discussed earlier is panel design and the associated spreading error. The impact of spreading error on high‐dimensional data can be significant, potentially leading to erroneous cell splitting into distinct clusters and resulting in biologically meaningless outcomes. An important step involves data preprocessing [35], which includes applying a quality‐control cleaning algorithm, such as flowAI [49] or PeacoQC [50], followed by data transformation. Notably, comparative studies have shown that PeacoQC outperforms flowAI in filtering various types of anomalies. PeacoQC identifies density peaks per channel and eliminates low‐quality events based on their position in the isolation tree and their mean absolute deviation distance from these peaks. It also incorporates quality‐control measures based on signal stability [50].

Subsequent data analysis steps involve normalizing the data before applying clustering and dimensionality reduction techniques, which can be accomplished using tools such as CytoNorm or SwiftReg. After normalization, clustering and dimensionality reduction can be performed, followed by downstream statistical evaluation [35]. Currently, cloud‐based software platforms such as OMIQ, Cytobank, CellEngine, and Cytolution support these processes, providing users with step‐by‐step guidance throughout the workflow. Notably, no prior programming experience—such as knowledge of R or Python—is required, and three of these platforms (OMIQ, Cytobank, and Cytolution) also offer automated gating capabilities.

**FIGURE 3:**
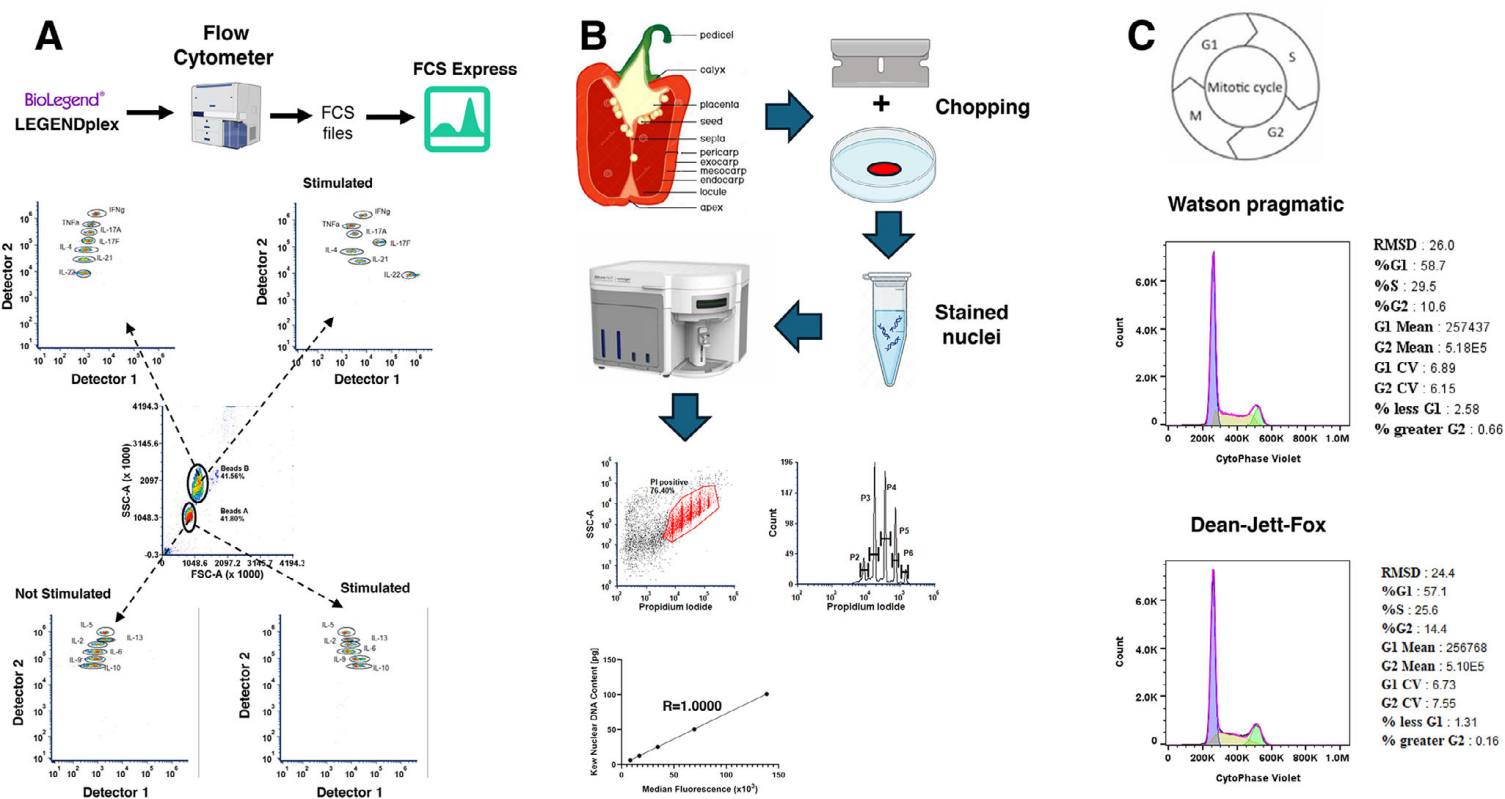
This figure provides a graphical outline of the analytical process for two common flow cytometry assays. (A) Demonstrates how multiplexing can be achieved by using labeled beads. In this case, multiple cytokines can be measured. In (B) and (C) are shown applications of DNA analysis. (B) Demonstrates endoreduplication in plant development, where somatic cells undergo successive rounds of genome replication (S‐phase) without mitosis. Consequently, these cells replicate their DNA repeatedly without division, resulting in polyploid cells that contain an increased number of chromosomes within a single nucleus. (C) Shows two analytical models for cell cycle analysis, the Watson pragmatic and Dean‐Jett‐Fox models.

The prevailing trend in flow cytometry data analysis is shifting toward automated, efficient processes to expedite biomarker discovery and disease surveillance. In this context, additional tools for automated data analysis include METAFlow and terraFlow [51, 52]. Both utilize proprietary AI‐driven technologies that perform multidimensional clustering of flow‐cytometry data through advanced unsupervised algorithms for automated biomarker discovery (MetaFlow) or conduct comprehensive searches for disease‐associated cell populations, presenting results in an easily interpretable format (terraFlow). Additionally, Ozette offers several AI‐driven tools for flow cytometry analysis. Ozette Discovery facilitates automated cell population identification and annotation for single‐cell cytometry data, while Ozette Endpoints provides automated biomarker endpoint analysis. Ozette Assay‐to‐Insights integrates these analytical capabilities with Ozette's high‐parameter immunophenotyping panel to generate comprehensive immune profiling analyses [53]. Hema.to employs an artificial intelligence‐based platform for automated classification and population detection in clinical flow cytometry samples. The platform uses machine learning algorithms to classify B‐cell non‐Hodgkin lymphoma (B‐NHL) subtypes and distinguish them from non‐malignant samples. The company's AI‐based diagnostic decision support system for B‐NHL has received CE‐IVD (Conformité Européenne—In Vitro Diagnostic) certification under the EU In Vitro Diagnostic Regulation. A web‐based interface allows users to upload flow cytometry data for automated analysis and diagnostic report generation [54, 55, 56, 57, 58]. Figure 3 provides a simple overview of two key flow cytometry processes, immunophenotyping and DNA analysis.

### 3.2 DNA Analysis

Flow cytometry has long been employed for the analysis of DNA content and cell cycle. This flow cytometry technique is regarded as the second most prevalent application following immunophenotyping. Flow cytometry enables the measurement of DNA content, yielding two essential insights: DNA ploidy, which identifies the copy number of homologous chromosome sets, and the distribution of cells throughout the cell cycle. DNA analysis has demonstrated its utility in delineating abnormal DNA content (aneuploidy) in human hematological malignancies, including acute leukemia, myelodysplastic syndromes, lymphoma, and multiple myeloma [59, 60, 61]. Conversely, it has demonstrated limited predictive value in solid tumors and low‐grade tumors [62, 63]. In this case, doing a double stain with DNA and cytokeratin might be more useful for clinical purposes [64].

In plants, DNA cytometry is used to study genome size by analyzing parameters related to it, such as ploidy level, or by comparing the genome sizes of different cell populations. Among these applications, estimating ploidy level remains the most common use of flow cytometry in plant sciences, particularly for investigating endoreduplication during plant development [65, 66, 67]. Endoreduplication is a process in which somatic cells undergo successive rounds of DNA replication (S‐phase) without entering mitosis—a phenomenon observed in species such as *Capsicum annuum* (pepper), *Zea mays* (maize), *Solanum lycopersicum* (tomato), and *Arabidopsis thaliana* (thale cress). As a result, these cells replicate their DNA multiple times without division, leading to polyploid cells containing increased chromosome numbers within a single nucleus. Each round of endoreduplication doubles the nuclear DNA content, producing somatic cells with nuclei of 2C, 4C, 8C, 16C, and so forth [68].

Successful evaluation of DNA is contingent upon effective sample preparation, the technical configuration of the instrument, and thorough data analysis. The initial step involves isolating single cells or nuclei while minimizing debris and clumps. This can be achieved by properly configuring the instrument settings during cell acquisition or utilizing instruments with acoustic focusing, which allows for rapid cell acquisition without compromising data quality [68, 69]. Traditionally, DNA content measurements were performed using dyes that bind stoichiometrically to DNA, indicating that the amount of bound dye is proportional to the amount of DNA present. Commonly employed DNA‐binding dyes include Vybrant DyeCycle violet, FxCycle violet, propidium iodide (PI), 4′,6‐diamidino‐2‐phenylindole (DAPI), DRAQ7, Helix NP NIR, Hoechst Janelia Fluor 526 and 646, and CellPhase violet [70, 71].

Analysis of cytometry histograms using single‐dye binding to DNA indicates overlap between early S phase and G1, and between late S phase and G2/M. A key challenge in analyzing DNA histograms is identifying a reliable model to accurately estimate the extent of this overlap. Several mathematical models used for cell‐cycle analysis are integrated into third‐party software designed for flow cytometry data analysis. The Dean/Jett method, recognized as the gold standard for cell‐cycle modeling, was initially introduced in 1974 [72]. In 1980, Fox modified this approach to enhance its applicability to data with complex S‐phase distributions [73]. Seven years later, another model, the Watson Pragmatic (WP), was proposed [74]. The Dean‐Jett‐Fox (DJF) model is particularly effective in resolving complex S‐phase distributions through polynomial modeling, making it well‐suited for perturbed or synchronized samples. The WP model offers an efficient and straightforward approach for routine analyses, although it may not provide the necessary flexibility for atypical cell‐cycle profiles.

To summarize, the WP model makes no assumptions about the shape of the S‐phase distribution, whereas the DJF model assumes that the S phase can be represented by a second‐degree polynomial convolved with Gaussian distributions of varying widths across the S phase. However, it is unclear which model is more appropriate, as both have been shown to be unreliable in estimating the duration of the S phase of the cell cycle [75]. DJF and WP models are integrated into third‐party software, such as FlowJo (BD Biosciences) [76] and Flowlogic [77].

In contrast, the software package ModFit (Verity) builds a mixture model (Gaussians + S‐phase shape + debris + aggregates) and optimizes it using Levenberg–Marquardt to minimize the residuals between the model and the data [78]. Despite that flow‐cytometric analysis of cell DNA content is widely used for the estimation of cell‐cycle phase distributions, this analysis does not provide cytodynamic information such as cycle traverse rates and phase transit times. Therefore, bivariate analysis of the cell cycle can be more informative, as it can combine phenotype analysis with cell‐cycle characterization [19, 79, 80].

Another approach from Verity involves so‐called probability state modelling (PSM) [81]. It models the DNA‐content histogram as a mixture of probability distributions, each representing a distinct biological state of the cell cycle (G_0_/G_1_, S, and G_2_/M). Instead of simply fitting peaks, PSM treats each cell's measured fluorescence as a random variable drawn from the probability density function of its true state. Fixed DNA states (G_1_ and G_2_/M) are modeled as Gaussian distributions, while the S‐phase is represented by a continuous probability function spanning the replication range between them. Consequently, PSM supports quantitative analyses of cell‐cycle kinetics, including phase durations and transition probabilities. Notably, PSM‐based software such as GemStone (Verity) can automate the analysis process, enhancing objectivity and improving reproducibility [81].

### 3.3 EV Analysis

Extracellular vesicles (EVs) are lipid bilayer particles produced by all cellular organisms. They have been the focus of extensive research for more than two decades due to their critical role in biological systems such as intracellular communication and cargo transport. EVs make significant contributions to immunology by modulating innate and adaptive immunity, as well as microbial responses. Furthermore, EVs can act as biomarkers for a variety of physiological and pathological conditions, providing useful information for diagnosis and prognosis. Finally, EV engineering shows potential for developing EV‐based immunotherapeutics for a wide range of diseases [82].

EVs can be categorized by size into three groups: small EVs (diameter: 50–150 nm), medium EVs (200–800 nm), and large EVs (≥1000 nm), with small EVs comprising the majority of these populations [82]. Flow cytometry, unlike microscopy techniques, is a reliable, high‐throughput method for studying EVs, particularly the heterogeneity within their populations, which includes size, concentration, surface markers, and quantification. Furthermore, flow cytometry, by analyzing EVs in body fluids, enables non‐invasive diagnostic approaches that provide valuable insights into disease progression and treatment responses. However, the development of methodological recommendations and standardized procedures for sample collection, storage, EV isolation, instrument setup, reagent selection, EV measurement, and data reporting is now critical. These issues have been thoroughly addressed in numerous publications and discussed among the EV research community at various workshops [83, 84, 85, 86, 87, 88, 89, 90, 91].

Advances in flow cytometry instrumentation, software, and reagents have significantly improved our ability to detect, characterize, and analyze EVs; however, performance varies depending on instrument and calibration method. There have been several attempts to resolve this issue. For example, the development of customized instrumentation with near‐100% single‐fluorochrome detection efficiency, as well as the establishment of methods for vesicle sizing based on the fluorescence intensity of a membrane dye, provide a reference methodology for comparing instrument sensitivity and calibration methods in the characterization of extracellular vesicles across instruments with varying limits of detection (LODs) [92].

Previous research has emphasized the importance of calibrating and defining EVs above LODs for accurate cross‐platform comparisons. In this context, light scatter, fluorescence, and concentration calibration are effective in producing reliable data from various flow‐cytometry instruments, regardless of gain/voltage settings, flow rate, or instrument collection angle. However, these conclusions may ignore the implications of single‐molecule domains or fail to compare the effects of bead‐based and single‐molecule calibration. Recent research on this topic shows that the nominal LOD values determined by hard‐dyed bead calibration were lower than the actual values, highlighting the benefit of using single‐molecule flow cytometers as a reference instrument for side‐by‐side comparisons and signal validation [92].

### 3.4 Functional Analysis

Flow cytometry is a powerful tool for developing a variety of functional assays that provide important insights into cellular processes and behaviors. These assays focus on the functional characteristics of cells rather than their phenotypes, and involve stimulating cells with specific agents or conditions, followed by measuring the changes in cellular parameters using fluorescent probes or dyes. This method allows researchers to observe cellular processes and responses in real time, making flow cytometry an effective tool for cell biology and immunology research [93].

Essential functional assays measurable via flow cytometry include cell viability and health (apoptosis detection, oxidative stress assessment, and overall cell viability), cellular processes (cell proliferation, phosphorylation, evaluation of intracellular parameters, endocytosis assessment, and detection of both extracellular and intracellular cytokines, in addition to immune cell activation), and intracellular parameters (membrane potential, intracellular calcium concentrations, and pH), alongside receptor occupancy assays. These techniques have been employed for numerous years and are thoroughly documented in the existing literature [94, 95, 96, 97, 98]. In this context, we will focus on the latest advancements in monitoring cellular functions, including cellular barcoding, cell metabolism, and analytical approaches for analyzing complex systems biology.

#### 3.4.1 Multi‐Pass Flow Cytometry

LASE Innovation Inc. has developed LP Cell Barcoding, a technology that allows for highly multiplexed barcoding of individual cells. The basic idea is to stain cells with laser particles (LPs), which are nano‐ to micron‐sized particles that emit narrowband infrared light (1000–1700 nm). A multi‐pass flow cytometer has been developed based on Beckman Coulter's commercial CytoFlex flow cytometer, with the functionality to read LPs and collect all post‐analysis cells enhanced. The cells are then stained and destaining (photobleached) sequentially, followed by measurement cycles.

In contrast, LP barcodes remain intact across analysis cycles, allowing for cell tracking and data integration into a single dataset. This technology allows researchers to measure a large number of markers, effectively overcoming the limitations of current traditional instruments. Thus, the combination of cellular barcoding and multi‐pass flow cytometry simplifies complex staining panels into fewer measurements, lowering complexity and minimizing spillover spreading errors. Furthermore, this technology goes beyond single‐time‐point cell measurements, allowing for detailed analysis of the downregulation and upregulation of key biomarkers across different experimental conditions. This method, also known as time‐resolved flow cytometry, has significant potential for use in precision medicine and the development of potency assays to investigate the effects of various drug candidates on cell function [99].

#### 3.4.2 Cell Metabolism Analysis

Flow cytometry is a valuable technique for analyzing cell metabolism at the single‐cell level, providing essential insights into metabolic diversity within complex cellular populations. Historically, flow cytometry has primarily focused on assessing mitochondrial potential or mitochondrial mass in activated cells using specific mitochondrial probes, including DiOC6, TMRM/TMRE, JC‐1, and various MitoTrackers (such as Green FM, Deep Red 633, and Red 580). In recent years, several novel applications have emerged, including Met‐Flow (metabolic enzyme and transporter analysis, fluorescent metabolite detection, and SCENITH single‐cell metabolic profiling in transient inhibition of metabolism) and RNA flow cytometry [100, 101].

Met‐Flow is a high‐parameter flow cytometry technique that uses antibodies to detect key metabolic proteins in immune cells. This method allows for the simultaneous measurement of several metabolic pathways, including glycolysis, the pentose phosphate pathway, the TCA cycle, and fatty acid metabolism, as well as the detection of metabolic remodeling in response to stimuli or environmental changes [101, 102]. SCENITH enables the ex vivo analysis of global metabolic profiles within complex cell populations, while simultaneously facilitating the phenotyping and metabolic profiling of multiple cell types [103]. RNA flow cytometry integrates transcriptional analysis with metabolic profiling, offering insights into cellular function [104]. Furthermore, flow cytometry can analyze cell metabolism at the single‐cell level by assessing nutrient uptake efficiency. For instance, glucose uptake can be evaluated by analyzing glucose transporter (GLUT) expression in conjunction with glucose incorporation, using specialized fluorescent probes such as 2‐NBDG or 6‐NBDG [100]. Additionally, flow cytometry can measure fluorescent metabolites and cofactors, facilitating the analysis of NAD(P)H and FAD levels to evaluate the cellular redox state [105]. While traditional flow cytometry has demonstrated success in analyzing these metabolites [105], it is believed that incorporating fluorescence lifetime measurements into either customized flow cytometry instruments or commercial systems can yield more precise results [106, 107, 108, 109, 110, 111].

#### 3.4.3 Data Analysis Approaches for Monitoring Cell Function

Over the last two decades, numerous analytical tools have been developed, including t‐SNE, UMAP, FlowSOM, Citrus, and Phenograph, among others. The literature [112, 113, 114, 115, 116] contains detailed descriptions of these tools, which are also included in various commercial software packages such as Cytobank, OMIQ, CellEngine, and Cytolution. Additionally, there are free, lesser‐known tools, such as the simplified presentation of extremely complex evaluations (SPICE) [117, 118] and Floreada.io [119]. SPICE is a powerful data‐mining and visualization tool that was specifically designed for analyzing complex flow cytometry datasets, and it is especially useful for investigating immune‐cell polyfunctional profiles. Floreada.io is a free software platform that provides functionality similar to FlowJo or FCS Express, including essential tools for cell analysis and research.

For several years, our laboratory at Purdue University has also created specialized software packages for both basic and extensive cell analysis, including Xploid (DNA ploidy analysis), PlateAnalyzer (multiplexed cell analysis), and CytoSpec (flow cytometry data analysis) [120, 121].

Recent advances in machine learning techniques have fueled the development of numerous new software packages. They result in an increased ability to use cytometry data to identify subtle differences between healthy and diseased cells [122, 123]. In another area, TimeFlow was recently introduced as a novel method for pseudotime computation in the analysis of multidimensional flow‐cytometry data. This method enables researchers to gain a better understanding of cellular differentiation processes and population dynamics in flow cytometry studies [124]. All existing and emerging methods have the same goal: to improve the accuracy, efficiency, and depth of flow‐cytometry data analysis, allowing researchers to gain more insights from complex datasets and facilitating the discovery of new cell populations and biomarkers.

### 3.5 HIV Diagnosis

Flow cytometry has long been regarded as an essential instrument in HIV research [125, 126, 127, 128, 129]. Recent advancements in flow cytometry for HIV detection encompass the incorporation of imaging flow cytometry for the visualization and quantification of HIV particles within cells, the identification of intracellular HIV p24 antigen in T cell subsets, and high‐throughput “flow virometry” for single‐virion analysis [130]. These innovations improve diagnostic and research capabilities beyond conventional CD4+ T cell counting, facilitating a more accurate evaluation of HIV persistence, immune activation, and therapeutic effectiveness.

### 3.6 Microbial Analysis

The application of flow cytometry (FC) in microbial research dates to the 1970s [131]. Over the years, FC has become an increasingly valuable tool for bacteria characterization in various fields, such as oceanography, host‐microbiome interactions, susceptibility testing, and monitoring microbial activity and metabolism, as well as in food safety and probiotic production [132, 133, 134, 135, 136]. The utility of FC is highlighted by its ability to assess total bacterial cell counts, evaluate bacterial viability, identify bacterial species, conduct biological functional studies on bacteria, and characterize their physiological changes in response to environmental conditions [137, 138, 139, 140, 141, 142, 143].

The assessment of bacteria can be effectively characterized through various techniques, including nucleic‐acid staining, sequence‐specific nucleic‐acid probes, fluorogenic substrates, efflux‐pump staining, reactive dyes, or immunofluorescent staining [144, 145, 146, 147, 148]. Microbial quantification is carried out using DNA stains such as PicoGreen, SYTO dyes, SYBR dyes, SYTOX dyes, and acridine orange, in conjunction with a fluorescent bead standard of known particle concentration or volumetric flow rate [148]. While these dyes exhibit weak fluorescence in solutions, they become highly fluorescent upon intercalation with DNA. In practice, bacterial enumeration has been successfully implemented across various samples, including drinking water, soil, activated sludge, vaginal swabs, and milk, as well as in the production of probiotics [148].

The bacterial viability test is typically conducted using the commercially available LIVE/DEAD BacLight Bacterial Viability Kit, which utilizes a combination of SYTO‐9 and propidium iodide (PI). SYTO‐9 is a permanent dye that stains both viable bacteria and those with damaged membranes, whereas PI penetrates only bacteria with compromised membranes and has a greater binding capacity for double‐stranded DNA compared to SYTO‐9. When used in combination, SYTO‐9 and PI provide preferential staining for viable and dead bacteria, respectively. Other combinations of dyes for bacterial viability assessment reported in the literature include thiazole orange (TO) and PI, SYBR Green I and PI, and carboxy fluorescein diacetate and PI [148].

Dyes can also be used to distinguish between bacterial types. Gram‐positive and Gram‐negative bacteria, for example, differ in terms of cell envelope structure. Gram‐negative bacteria have a peptidoglycan layer sandwiched between their outer and inner membranes, whereas Gram‐positive bacteria have a thick peptidoglycan layer surrounded by an inner membrane. The combination of hexidium iodide (HI) and SYTO‐9 allows for the distinction between Gram‐negative and Gram‐positive bacteria. However, it is important to note that, while SYTO‐9 can stain both Gram‐positive and Gram‐negative bacteria, its combination with hexidium iodide allows for more precise differentiation. Nonetheless, this technique does not appear to be accurate for uncultivated samples. As a result, a modified approach is recommended, which involves staining bacteria with Oregon Green‐conjugated wheat‐germ agglutinin (WGA) and HI. In this context, WGA specifically stains Gram‐positive bacteria, while HI binds to the DNA of all bacteria following permeabilization with EDTA and subsequent incubation at 50°C for 15 min. This methodology has been validated for milk samples inoculated with *Staphylococcus aureus* and *Escherichia coli*, without the need for precultivation. Additionally, certain DNA stains exhibit differential binding to specific base pairs, such as adenine and thymine (i.e., DAPI and Hoechst 33342) or guanine and cytosine (i.e., chromomycin, mithramycin, and olivomycin). The combination of these stains enables bacterial classification based on the measurement of % GC content [147, 149].

Bacterial responses to environmental variables such as temperature, pH, oxygen, chemicals, and antibiotics can be difficult to quantify using traditional methods due to transient changes that may not be directly related to genetic factors. For example, antimicrobial resistance is based on the overexpression of efflux pumps, which can expel a variety of drugs from bacteria, resulting in multidrug resistance. Using a substrate for this pump, such as ethidium bromide, the pump's activity and expression level can be measured using flow cytometry. Conversely, measuring membrane permeability, esterase activity, or redox activity has practical applications in assessing bacterial responses to physical or chemical stress, particularly in ensuring that food preparation processes are free of contamination by *L. monocytogenes* and *B. cereus* [148].

Finally, different bacteria's biological functions can be monitored using fluorescent protein expression (gene reporters) or enzymatic activity measurements. In this context, GFP was used to monitor development cycle of *Chlamydia trachomatis*. Fluorogenic substrates are non‐fluorescent until they enter cells and are converted into fluorescent products via enzymatic reactions. For example, 5‐cyano‐2,3‐ditolyl‐tetrazolium chloride (CTC) was used to study bacterial respiration at the single cell level. Dehydrogenase reduces CTC, producing a product with red fluorescence whose intensity corresponds to the rate of respiration [148].

Not all flow cytometers are equipped to effectively detect bacteria owing to variations in technical configuration.

Bacteria typically range from 0.2 to 10 µm, requiring careful consideration of multiple factors when designing experiments. To reduce background noise from sub‐micron particles in the sheath fluid, use filtered fluid before analysis. Furthermore, bacterial efflux systems can rapidly expel dyes (such as propidium iodide), which can lead to false negatives in viability or membrane integrity assays. Propidium iodide uptake can be compromised in some cases (as seen with *Bacillus* spores), and *Legionella* may produce variable scatter signals [148].

Furthermore, limitations in probe selection based on fluorescent emission can result in spectral overlap. A spectral sorter specifically designed for bacterial work, such as BigFoot (Thermo Fisher Scientific), provides a partial solution to this problem; however, meticulous preparation both before and after the experiment is critical for ensuring data reproducibility, as discussed in detail elsewhere [150]. Among traditional flow cytometers, suitable instruments for studying bacteria include the Cytoflex, BD Symphony A1 (equipped with a small particle detector), and Apogee MicroPlus, which demonstrate particle detection capabilities of 80, 90, and 70 nm, respectively [148, 151]. Another approach documented in the literature involves label‐free detection of bacteria utilizing the autofluorescence of their cell walls [152].

Ongoing advancements in dye chemistry, microfluidics, and machine learning may aid in bacterial characterization via flow cytometry, particularly in environmental and clinical settings. Elastic light scattering technology was recently introduced to identify foodborne pathogens. When combined with spectral cytometry and machine learning, this technique provides a quick method for detecting and identifying bacteria [153].

## 4 Cell Sorting

Cell sorting involves the isolation of specific cell populations from heterogeneous samples based on their physical, chemical, or biological characteristics. This technique plays a crucial role across various fields, including immunology, cancer research, stem cell therapy, and microbiology [154, 155, 156]. The process can be carried out using a range of instruments and technologies, such as fluorescence‐based methods [157], label‐free approaches [158], and microfluidic systems [159].

Mack Fulwyler Field invented the first flow sorter in 1965 [160, 161]. He combined inkjet technology, first developed by Richard Sweet in 1964 [161], with the Coulter principle, which detects changes in electrical impedance as cells pass through an orifice. This innovation enabled the physical separation of individual cells based on their characteristics, resulting in the development of the first cell sorter and laying the groundwork for modern flow cytometry instruments [160]. Fulwyler's invention influenced subsequent developments, such as fluorescence‐activated cell sorting (FACS), which was pioneered by Herzenberg, Bonner, Sweet, and Hulett in the late 1960s. FACS incorporated fluorescence detection into Fulwyler's sorting principles, allowing for multiparametric cell analysis using surface markers or intracellular properties. In the early 1970s, this technology was commercialized by Becton Dickinson Immunocytometry Systems, evolving into what are now referred to as electrostatic sorters, which operate based on high pressure and electrostatic deflection [156, 159].

### 4.1 Electrostatic Sorters

Electrostatic sorters have proven to be effective and reliable instruments for sorting various types of cells. These devices enable the simultaneous collection of four or six populations through differential stream charging [156]. Electrostatic sorters are widely utilized and produced by various manufacturers worldwide, including Beckman Coulter (Cytoflex SRT), Becton Dickinson (FACS Aria III, FACS Aria Fusion, FACS Symphony S6), Biorad (S3e sorter), Cytek (Aurora CS), and Thermo Fisher Scientific (BigFoot) [159]. Among these, the Aurora CS and BigFoot are classified as spectral cell sorters; however, both traditional systems and spectral models employ the same cell‐sorting technology [156]. While electrostatic sorters are highly robust, their use can negatively affect cell health and function owing to factors such as nozzle diameter, sheath and sample pressure, the frequency and duration of stream drop generation, and the time taken for cells to travel from the laser intercept to the drop‐generation point [156]. Specifically, sorting with electrostatic cell sorters has been shown to impact the redox state and cellular metabolome [162, 163], as well as cell viability [164] and minimal gene expression changes across different populations of mouse mammary cell subsets [165]. Conversely, other studies indicate that factors such as nozzle size, pressure, UV exposure, and instrument type have minimal effects on gene expression [166].

It is also worth noting that, unlike microfluidic‐based cell sorters, electrostatic sorters are primarily used in open‐air environments, necessitating the use of containment systems for samples classified as biohazard [156]. In addition, a study using spectral instruments shows that analyzing cells in one suspension buffer and sorting them into another can cause changes in fluorochrome emission and/or excitation profiles. These changes may cause differences between the spectral signatures of sorted cells and single‐stain controls, potentially leading to unmixing errors [167].

### 4.2 Microfluid‐Based Cell Sorters

Microfluidic systems use microscale channels and chambers to sort cells gently and precisely. One significant advantage of this technology is the reduction of sorter‐induced cellular stress, which aids in recovery and cell viability. Furthermore, these systems use disposable, closed cartridge designs, which improve usability and sterility while limiting aerosol production [156, 159, 168, 169]. Researchers can also use custom fluids, such as culture media, and disposable chips are cost‐effective, eliminating the need for ongoing maintenance. However, some limitations of this technology include the limited number of detectors and lasers, as well as the speed of cell sorting [156]. Recent advancements in microfluidics technology have resulted in notable cell sorters such as the MACS Quant Tyto sorter (Miltenyi), On‐chip Sort (On‐Chip Biotechnologies), and Wolf Cell Sorter (Biosystems) [159].

### 4.3 Imaging‐Based Sorting

Image‐activated cell sorting integrates high‐speed imaging and sorting techniques to separate cells based on their visual and subcellular characteristics. For example, the BD FACSDiscovery S6 combines spectral analysis with real‐time imaging, allowing for comprehensive multiparametric phenotyping, morphology characterization, and sorting based on marker localization within cells. This capability is especially useful in complex experiments involving intricate biological systems [170, 171]. Other image‐activated sorters include iIACS 2.0, an AI‐powered microfluidic IACS system dubbed intelligent IACS. Although iIACS is not commercially available, it is described in a methodological paper [172]. We will discuss the use of imaging in cytometry in subsequent sections.

### 4.4 Label‐Free Cell Sorting

While typical IACS approaches use fluorescence imaging, the computational sorting and mapping of single cells (COSMOS) method does not include fluorescence detection; instead, it uses morphometric data from brightfield images. As a result, this label‐free method is ideal for difficult samples, such as patient samples, by providing insights into different cell types or states [173]. Technically, COSMOS is a cloud‐enabled platform that facilitates real‐time cell imaging, analysis, and sorting through deep learning–based morphology representations. It includes a comprehensive atlas of annotated single‐cell images, a collection of deep‐learning models capable of classifying label‐free brightfield images, and fluidics hardware designed to isolate target cells based on model classifications. This platform has proven effective for visualizing significant morphological distinctions across biological samples and for accurately discriminating and enriching specific cells of interest without the use of labels [174].

## 5 Advances in Flow Imaging and Label‐Free Cytometry

The original concept of imaging in flow was tried nearly 50 years ago with a specialized movie camera and a high‐speed trigger device [175]. This device had a maximum capacity of 150 images per second and was limited to 4000 images based on a 30‐m length of film in the camera cassette. The instrument was successful in detecting singlets, doublets, and triplets, but that was the extent of its capabilities. An innovative concept Field [176], that George Dubelaar and colleagues later developed into the CytBuoy, an ocean‐going flow cytometer [177]. Over the past 25 years, this technology has advanced scatter, shape, fluorescence, and AI to a remarkable level [178, 179, 180, 181, 182].

To a large extent, this technology defined the potential for a combination of flow and imaging, though it remained focused on a specific area of marine sciences, which we believe was beneficial to marine sciences but detrimental to the rest of the cytometry community.

Some 20 years after the original concept, a much more practical solution that was more relevant to and focused on eukaryotic cell systems was provided with ImageStem technology, and it has remained until recently the only technology capable of including cellular imaging in combination with traditionally collected flow cytometry parameters such as scatter and fluorescence [183]. With the commercialization of ImageStream, the value of combining imaging and flow has significantly enhanced the field [183]. Despite the images' low resolution, the combination proved extremely useful in a variety of scientific fields. When this technology was released, the cytometry community was particularly interested in apoptosis. The ImageStream was able to quickly distinguish between necrotic and apoptotic cells in the same system, which could not previously be done.

Over the past decade, a number of attempts at producing combination flow‐imaging approaches have emerged. Some have used the principle of light‐sheet fluorescence microscopy to measure water samples [184], and more recently, a faster instrument focused on phytoplankton was developed [185]. Conversion of conventional instruments into combined flow imaging has also been achieved using the CytPix [186] or instruments such as the Flowcam [187]. The most recent imaging instrument is the S8, which combines virtually all the possible measurement modes in flow cytometry [170], including the possibility of sorting on images. These are certainly valuable advances, but the relatively low image quality remains the biggest issue, and it may take some time before the image resolution is sufficient to really advance this combination. It will be interesting to see how AI can leverage this to expand the technology.

Ghost cytometry, developed by ThinkCyte, facilitates label‐free cell sorting by combining high‐resolution morphological profiling with artificial intelligence, rather than relying on fluorescent labels or molecular markers [188].

In short, it examines optical diffraction and scattering signals as cells pass through patterned light sources. The resulting detailed morphological waveforms are compressed and processed in real time by machine learning models trained to recognize specific cell types or functional states. Supervised and unsupervised machine learning algorithms interpret these high‐content diffraction signals, assigning computational “labels” to cells based on their distinct morphological characteristics. These solutions are implemented in ThinkCyte's VisionSort platform, which allows for the rapid characterization and sorting of cells at speeds of up to 3000 events per second. Using morphology‐based labels, this platform can gate, separate, and collect cell populations without inducing cytotoxicity, while also reducing costs associated with molecular staining and maintaining live, untouched cells for downstream functional assays and therapeutic applications [189].

## 6 Mass Cytometry: An Alternative Detection Paradigm

The spectral versus polychromatic debate represents competing strategies within fluorescence‐based detection, but a fundamentally different approach exists: replacing fluorescence entirely with mass spectrometry. Mass cytometry (also known as CyTOF, for Cytometry by Time‐Of‐Flight) sidesteps the spectral overlap problem by labeling antibodies with heavy metal isotopes rather than fluorophores. Because each isotope has a distinct atomic mass, signals are inherently orthogonal. This elegant solution enables measurement of 40+ parameters simultaneously without compensation or unmixing [190, 191, 192].

Mass cytometry has experienced significant growth over the past decade, driven primarily by applications in immunology, oncology, personalized medicine, and translational research. Standard BioTools (formerly Fluidigm) established the field with their CyTOF platform and has remained the dominant provider of instrumentation and software.

In 2021, a competing platform entered the market: the Polaris Starion system [193]. Like the CyTOF, the Starion enables analysis of over 40 parameters simultaneously through mass spectrometry detection. However, the Starion system introduced an innovation in sample throughput through 120‐sample barcoding technology. This approach utilizes unique combinations of metal isotope tags to individually label each sample prior to pooling, enabling analysis of up to 120 samples concurrently in a single run, followed by efficient parallel processing and computational demultiplexing of the data. This multiplexing capacity addresses one of mass cytometry's traditional limitations: relatively low single‐sample throughput compared to fluorescence‐based systems.

The fundamental trade‐offs between mass and fluorescence‐based cytometry are instructive. Mass cytometry offers superior parameter depth without spectral constraints, making it ideal for comprehensive immunophenotyping and discovery applications. However, it sacrifices several capabilities: cells cannot be sorted for downstream analysis (they are destroyed during ionization), acquisition rates are substantially lower (∼500 events/second vs. 50 000+ for fluorescence systems), and the technology requires specialized expertise and infrastructure. Fluorescence‐based systems, whether polychromatic or spectral, maintain advantages in throughput, sorting capability, and sensitivity for low‐abundance antigens.

Perhaps most significantly, mass cytometry lacks spatial. This limitation distinguishes flow‐based mass cytometry from imaging mass cytometry (IMC), a related but distinct technology that ablates tissue sections to generate spatially‐resolved, high‐parameter images. The two approaches (flow‐based CyTOF and tissue‐based IMC) are complementary tools within the mass cytometry ecosystem.

The field has yet to fully assess the performance of the Starion system. At the time of this writing, no English‐language peer‐reviewed papers with actual experimental data from the Starion platform have been published. This absence of independent validation is notable given the system's 2021 commercial launch, though it may reflect the lengthy timeline between instrument installation, experimental execution, and publication in the mass cytometry community.

**FIGURE 4:**
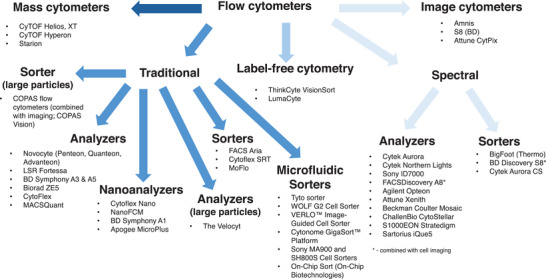
There are many types of cytometers available mostly focused on feature differences. These range for very high cost instruments (Mass cytometers, cell sorters), to low cost analyzers.

Mass cytometry occupies a specific niche in the single‐cell analysis landscape: where absolute parameter depth is prioritized over throughput, sorting capability, or real‐time analysis. The technology demonstrates that the parameter ceiling is not fundamental to single‐cell cytometry but rather specific to fluorescence‐based detection. Whether mass cytometry or spectral fluorescence ultimately proves more enabling for biological discovery likely depends on the questions being asked—a theme that recurs throughout the evolution of cytometry technologies. Figure 4 provides a comprehensive overview of all current cytometers covering the above technologies.

## 7 Challenges of the Current Technology

### 7.1 Labels and Antibodies

The fundamental principles of flow cytometry have changed little in the first 40 years of its use. Detectors used PMTs, optical systems were moderately basic, and light sources were almost entirely commercial continuous‐wave (CW) lasers. In the polychromatic world, nearly every instrument was a copy of another. Incremental changes were replicated across manufacturers. Barcoding [194], when implemented on one brand, was quickly spread to all instruments. Cameras for droplet breakoff, more lasers, more PMTs, and basically any useful feature were incrementally added across the instrument range. Some focused on expanding the number of available parameters [8, 10, 195], while others concentrated on high‐throughput sampling [196, 197].

The iterative increase in the number of desired labels drove the increase in the number of PMTs and lasers. Systems became increasingly complex and costly. At the same time, the chemistry of fluorescent dyes became an important issue. Much of the credit here likely goes to the late Dick Haugland, who founded Molecular Probes in 1975, transforming it from a garage startup into the definitive supplier of fluorescent reagents for the life sciences. His creation of the Handbook of Fluorescent Probes in 1985 [198] provided researchers with an unprecedented catalog and guide to fluorescent probe chemistry. Both the company and the handbook became essential resources that, over the next several decades, helped advance probe chemistry, expand the available fluorophore palette, and democratize access to specialized reagents that had previously been available only to chemists who could synthesize them. The company's impact on flow cytometry cannot be overstated: nearly every multicolor experiment relied on Molecular Probes reagents. Following Haugland's vision through acquisition by Invitrogen (2003) and subsequent integration into Thermo Fisher Scientific, the Molecular Probes product line remains central to fluorescence‐based research.

The development of hybridoma technology for monoclonal antibody production [4] created an opportunity that flow cytometry companies seized rapidly. Ortho Diagnostic Systems was among the first to commercialize fluorochrome‐conjugated monoclonal antibodies specifically for flow cytometry applications in the late 1970s, quickly followed by Becton Dickinson and Coulter Corporation in the early 1980s. The commercial availability of standardized, pre‐conjugated reagents proved transformative: laboratories no longer needed expertise in antibody conjugation chemistry to perform multicolor immunophenotyping. This democratization of the technology drove the rapid expansion of flow cytometry into mainstream immunology and clinical diagnostics. Multiple specialized antibody companies, including those that would later become PharMingen (now BD Biosciences), Caltag (later Invitrogen), and others, emerged to supply the growing market with flow cytometry‐optimized reagents.

### 7.2 Data Standardization

As instruments proliferated and data exchange between laboratories became necessary, the lack of a standardized file format created obvious problems. Different manufacturers used proprietary formats, making it difficult to analyze data from multiple instrument types or share data between laboratories. In the early 1980s, driven by the International Society for the Advancement of Cytometry (ISAC), a committee was established to define a universal standard [199, 200]. The resulting Flow Cytometry Standard (FCS) file structure (first released in 1984 and subsequently updated through FCS 3.1) represents one of the most successful standardization efforts in biological instrumentation. Remarkably, all manufacturers adopted the format voluntarily, and it has served the field through four decades of dramatic technological evolution. The FCS standard's longevity demonstrates that when standardization serves obvious collective interests, even competitive manufacturers will cooperate.

### 7.3 Demultiplexing

Perhaps no technical issue in flow cytometry has generated more confusion, tutorials, and frustration than compensation [201]. This persists with spectral cytometry and an ambiguous differentiation between compensation and unmixing that many practitioners overlook. These terms are routinely used interchangeably because they describe fundamentally similar operations, though with key distinctions in their mathematical formulations. Because of the specific normalization choices in the mixing matrices, compensation removes estimated spillover contributions from one fluorochrome to another. In contrast, unmixing follows the mathematical framework from remote sensing and spectral imaging to reconstruct true signals from overlapping spectra. The mathematical difference in the model is very subtle but consequential: how the mixing matrix is normalized and presented determines whether the algorithm performs true spectral deconvolution or simply subtracts spillover estimates.

This distinction has been obscured by imprecise terminology and, more problematically, by vendor implementations that claim to “unmix” data while actually performing compensation on increasingly larger datasets. The transition from polychromatic to spectral cytometry promised to replace subjective operator adjustment with objective computational approaches. Instead, it transferred the subjectivity from manual compensation matrix creation to algorithmic choices that are rarely documented transparently. Different spectral platforms implement fundamentally different “unmixing” strategies, yet these differences are seldom disclosed in sufficient detail for users to understand fully their data processing pipeline.

The practical consequence is a reproducibility problem that persists across both polychromatic and spectral cytometry: two analysts processing the same dataset may produce different results depending on mixing matrix normalization choices, quality control settings, and reference spectra selection. While such variation may not alter biological conclusions in robust experiments, it undermines claims of quantitative precision and complicates cross‐platform or cross‐laboratory comparisons.

This situation warrants a thorough examination, ideally a manuscript that systematically compares vendor implementations, clarifies terminology, and proposes standards for transparent reporting of unmixing approaches. Such research would have to address the unsettling fact that most cytometry companies misrepresent their unmixing algorithms, either through oversimplification in user documentation or through marketing claims that gloss over mathematical details. Users deserve to know how their data is processed, especially since spreading errors and other artifacts rely heavily on the unmixing strategy. Also, having a thorough understanding of unmixing would allow for better decisions during panel design.

For present purposes, we note that spectral cytometry has not solved the compensation problem but rather transformed it into a more complex unmixing challenge that remains incompletely standardized and often misunderstood by both users and, apparently, some vendors.

### 7.4 Demise of Polychromatic Cytometry

It could be argued that the most recent challenge to the field has been the gentle decline of polychromatic cytometry. Having reached its practical limit of around 30 fluorescent probes in simultaneous parameter collection, PC now finds itself increasingly relegated to simpler applications—a transition from the cutting edge to comfortable senescence. The field has moved on, embracing spectral flow cytometry with the enthusiasm typically reserved for technologies that double one's analytical capacity. As current instruments approach retirement age, their replacements arrive bearing spectral capabilities.

If we may be forgiven the metaphor, polychromatic cytometry served admirably for five decades, pushing the boundaries of multiparameter analysis as far as the visible spectrum and optical physics would allow. Its limitations were not failures but rather the natural constraints of its design—much as aging is not a disease but an inevitable consequence of biology. Spectral cytometry simply recognized that the spectroscopic paradigm, combined with computational unmixing, could circumvent these fundamental limitations. The transition is inexorable, though not without nostalgia for a technology that enabled much of modern immunology.

## 8 Recent Progress in Spectral Approaches

### 8.1 Instruments

Sony introduced the SP 6800, the first commercial spectral cytometer, in 2014, with three lasers, 66 channels, and automated alignment using CoreFinder technology. In late 2017, Cytek Biosciences introduced a spectral cytometer with three lasers and 38 avalanche photodiode detectors (APDs), which was later upgraded to five lasers and 64 detectors. In 2019, Sony launched the ID7000 spectral cytometer with seven lasers and 184 detectors, featuring grating‐based spectral dispersion, a higher cell‐acquisition rate (40 000 cps), an automated plate loader, and improved fluorescence sensitivity. Two years later, BD Biosciences introduced the Symphony A5 SE spectral analyzer with five lasers, 48 square PMT detectors, a similar cell‐acquisition rate, but slightly lower fluorescence sensitivity than the ID7000.

Agilent and Thermo Fisher Scientific released their respective spectral flow cytometers, the NovoCyte Opteon and Attune Xenith, in 2024. Agilent's instrument has five lasers, 73 APD detectors, fixed alignment, a syringe pump for sample delivery, an automatic sample loader, a high cell acquisition rate (100 000 CPS), and software with a pre‐loaded library of over 900 fluorochromes and dyes. The instrument is relatively new, with limited data on staining panel performance, but preliminary tests indicate that it can accommodate at least a 45‐color staining panel. The Attune Xenith from Thermo Fisher Scientific is equipped with six lasers and 51 PMT detectors.

Beckman Coulter recently demonstrated a spectral component called the Mosaic, which can be added to an existing CytoFlex to measure a 40‐color staining panel. With its use, a standard flow cytometer (Cytoflex LX with six lasers [UV, violet, blue, yellow, red, and infrared] capable of measuring up to 21 colors) can be converted into a spectral instrument by incorporating a novel 88‐channel spectral detection module. The instrument was shown to measure 40 previously reported markers in OMIP‐069 with fluorescence modifications. To address the signals' heteroskedasticity, an unmixing algorithm incorporating the Poisson noise model was used instead of the conventional least squares method.

All current spectral instruments in use in the United States are designated for research use only (RUO), which may contribute to their slow adoption in industrial settings. While these instruments are becoming more widely used in contract research organizations (CROs) and pharmaceutical companies, change adoption remains slow.

### 8.2 Sensitivity

As mentioned before, spectral flow cytometers differ fundamentally from conventional instruments in their approach to fluorochrome discrimination. Conventional cytometers use dichroic mirrors and bandpass filters to achieve optical separation of fluorochrome emission spectra, directing distinct, non‐overlapping wavelength regions to separate detectors. In contrast, spectral cytometers rely on computational unmixing algorithms to mathematically resolve signals from different fluorochromes post‐acquisition. This reliance on unmixing rather than optical separation enables some spectral cytometer designs to employ detectors that collect spectrally adjacent wavelength intervals across the entire available emission spectrum, without requiring optical gaps between detection windows.

This architectural approach raises the hypothesis that such spectral cytometers may capture a greater proportion of emitted photons compared to filter‐based systems. The continuous wavelength coverage in adjacent‐band designs could theoretically minimize photon loss that occurs in the spectral gaps between discrete detection windows in conventional instruments. If validated, enhanced photon collection efficiency could translate to improved signal sensitivity and measurement precision.

However, so far this hypothesis has not been empirically demonstrated through direct instrumental comparisons using standardized metrics such as signal‐to‐noise ratio, resolution index, or limit of detection. It is also important to note that not all spectral cytometers employ the adjacent‐band detection architecture; instrument designs vary, and this hypothesis applies only to those systems that do utilize a spectrally contiguous setup. Moreover, both spectral and conventional flow cytometers routinely generate data with sufficient signal intensity for reliable population identification and quantification in typical experimental applications, making the practical significance of any photon collection differences unclear.

Systematic comparative studies employing standardized fluorescent calibration beads, matched fluorochrome panels, and quantitative performance metrics are needed to test whether the adjacent‐band architecture translates to measurable advantages in analytical sensitivity or data quality.

### 8.3 Quantitative Cytometry

A long‐standing goal in flow cytometry is the standardization of fluorescence intensity measurements to enable quantitative comparisons across instruments, laboratories, and time points. While flow cytometry excels at enumerating biological particles and resolving differences in cellular physical properties, fluorescence intensity measurements are typically reported in arbitrary units that lack absolute meaning or cross‐instrument comparability.

Schwartz and colleagues pioneered efforts to address this limitation through the development of fluorescence calibration beads [202] and the concept of molecules of equivalent soluble fluorophore (MESF) [203, 204, 205, 206, 207, 208, 209, 210]. In partnership with the National Institute of Standards and Technology (NIST), this work established a traceable standard for converting arbitrary fluorescence units to standardized values representing equivalent numbers of fluorophore molecules [208, 209, 210]. The MESF approach has been successfully applied in clinical cytometry [204], and remains the reference method for quantitative fluorescence measurements [205, 206, 207].

Notwithstanding this established methodology, MESF‐based quantification has not attained widespread acceptance in research cytometry. Obstacles to implementation include the necessity for regular calibration with standards corresponding to experimental fluorochromes, increased time and complexity in experimental workflows, instrument‐to‐instrument variability in optical configurations necessitating distinct calibration, restricted commercial availability of MESF standards for certain fluorochromes, and the absence of standardized analysis workflows in commercial software.

Spectral cytometry architectures that eliminate adjustable optical filters from the emission path may reduce certain sources of measurement variability, potentially simplifying quantitative calibration. However, empirical data comparing quantitative reproducibility (e.g., coefficient of variation in MESF measurements) between spectral and conventional instruments across multiple laboratories and time points are needed to test this hypothesis.

## 9 The Next Generation—Cytometry 3.0

### 9.1 Reproducibility

The future of cytometry depends on rethinking instrumentation design to enable better quantitative standardization. Significant changes have already occurred over the last decade, with APDs largely replacing PMTs in many instruments. This transition has reduced instrument size and cost while improving certain performance characteristics. APDs demonstrate better linearity across extended dynamic ranges and more consistent detector response over time, both important factors for reproducible measurements. Most instruments still rely on numerous adjustable optical components, including bandpass filters and dichroic mirrors to direct light to detectors, while some use spectrometers that provide full‐spectrum coverage. The critical challenge ahead is developing technologies and protocols that enable true quantitative standardization, meaning traceable calibration to reference materials that produce comparable measurements across instruments, laboratories, and time points. Achieving this requires more than just improved detectors or optics; it demands rigorous implementation of calibration standards (like MESF beads), quality control procedures, and community adoption of reporting standards such as MIFlowCyt. If we can establish robust quantitative standardization, we gain reproducibility that minimizes instrument‐to‐instrument variability and enables meaningful data comparison, though biological variation and measurement uncertainty will always introduce some degree of variability. This is not an easy task, but standardized quantification is likely to be one of the most significant advances the field can achieve.

### 9.2 Excitation Systems

Another feature that will move us toward Cytometry 3.0 will be a complete rethinking of flow cytometer excitation systems. Currently, nearly all flow cytometers employ CW lasers that provide stable, constant illumination throughout data acquisition. While this design offers simplicity and proven reliability, it limits experimental flexibility. Developing dynamically controllable excitation systems, where individual lasers can be rapidly modulated in power, switched on or off, or triggered in sequence, could enable new experimental approaches currently unavailable in conventional flow cytometry. For example, pulsed high‐power laser excitation could trigger photosensitive molecular probes or initiate rapid physiological responses in cells during measurement, similar to photo‐activation techniques established in microscopy. Sequential laser activation could enable kinetic measurements during the brief transit time through the interrogation point, potentially revealing dynamic cellular responses. Conditional excitation, where downstream lasers are triggered only when upstream detectors identify specific markers, could reduce photobleaching and phototoxicity while enabling more complex multi‐parameter decision trees in cell sorting applications. However, implementing such systems presents substantial technical challenges: synchronizing laser modulation with microsecond‐scale cell transit times, maintaining measurement precision during power transitions, managing increased instrument complexity and cost, and developing appropriate controls and calibration protocols. These technical hurdles require systematic investigation before practical implementation.

### 9.3 True Single Photon Detection

The final enhancement to Cytometry 3.0 will involve the implementation of single‐photon counting detectors. While both conventional flow cytometry detectors and single‐photon counting detectors convert photons to photoelectrons via the photoelectric effect, the fundamental difference lies in how these photoelectrons are measured and processed.

Current flow cytometry systems use PMTs and APDs operating in analog integration mode—they collect and sum all photoelectrons generated during a measurement period into a continuous electrical signal proportional to the total light intensity. In contrast, single‐photon counting detectors operate in Geiger mode or photon‐counting mode, where each individual photon arrival generates a discrete, resolvable electrical pulse that can be counted separately. This allows the detector to count photons one by one rather than measuring their integrated intensity.

Single‐photon detector technology has been developing since the 1960s with the invention of single‐photon avalanche diodes (SPADs), building on earlier photomultiplier tube technology. Recent decades have seen significant advances in superconducting nanowire single‐photon detectors (SNSPDs) and other technologies, driven by applications in quantum optics, quantum computing, and quantum key distribution.

This same photon‐counting principle has been successfully implemented in advanced computed tomography scanners. Photon‐counting CT (PCCT) systems, also called spectral photon‐counting CT scanners, received FDA approval starting in 2021. These systems use semiconductor detectors (cadmium telluride or silicon) that can count individual X‐ray photons at extremely high flux rates (billions of photons per second per mm^2^) while measuring their energy, providing superior image quality with reduced radiation dose compared to conventional energy‐integrating CT detectors [211].

Photon‐counting detectors measure the energy of individual photons, which allows determination of their wavelength since photon energy and wavelength are inversely related (*E* = hc/λ). This energy‐resolving capability provides spectral information previously not possible with conventional energy‐integrating detectors. This represents a transformational change in CT imaging and has become feasible due to advances in semiconductor detector technology that can handle the extremely high photon flux rates required for CT scanning (billions of photons per second per square millimeter).

These technologies could bring significant advantages to flow cytometry, primarily through improved quantification and the potential for energy‐resolved detection. Single‐photon counting detectors are beginning to emerge in research flow cytometry applications [212].

Fluorescence lifetime measurements—the measurement of fluorescence decay kinetics rather than photon energy—have proven valuable in flow cytometry for several decades. This parameter measures how long a fluorophore remains in its excited state before returning to ground state, typically in the nanosecond range. Combined with phenotypic markers, fluorescence lifetime analysis enables evaluation of molecular environments, protein‐protein interactions (FRET), intracellular drug distribution, receptor kinetics, and cellular metabolism [213].

## 10 Conclusions

Flow cytometry has proven to be one of the most enduring technologies in the biological sciences. It has survived the molecular revolution that transformed many diagnostic approaches because of its unique ability to evaluate multiple parameters simultaneously in complex cell populations without requiring physical separation. The field emerged from a focus on engineering innovation that established a strong tradition of technology development spanning six decades. While currently heavily focused on immunophenotyping (enhanced by advances in spectral cytometry) improvements have extended across all aspects of the technology, from fluidics and optics to data analysis and standardization.

The cost per fluorescence parameter (“cost‐per‐color”) has decreased substantially compared to previous decades, making high‐parameter cytometry more accessible. This cost reduction is democratizing the field, enabling broader adoption and attracting new instrument developers offering advanced features at competitive prices. The substantial increase in computing capacity has transformed data analysis capabilities, drawing computational scientists and bioinformaticians to the field and driving significant advances in automated data analysis.

The critical question for cytometry is whether it will continue to drive biological discovery and clinical impact. To do so, the field must avoid becoming merely another service technology and instead remain focused on enabling scientific breakthroughs. In the early 2000s, the scientific impact of cytometry was recognized through the Kyoto Prize, awarded to the late Len Herzenberg in an acknowledgment of transformative scientific contributions, not just technical innovation. Contemporary examples include the development and monitoring of CAR T‐cell therapies, where flow cytometry provides essential analytical capabilities for both research and clinical implementation. Single‐cell analysis will remain scientifically essential for decades to come, but the field's continued relevance depends on addressing emerging biological questions: Can cytometry adapt to spatial biology and tissue contexts? Can it integrate with other single‐cell technologies? Can it achieve the quantitative standardization needed for precision medicine? The future of flow cytometry rests not on past achievements, but on its continued evolution to answer questions we have yet to fully articulate.

## Author Contributions

Conceptualization: J. Paul Robinson, Grzegorz B. Gmyrek, and Bartek Rajwa. Writing – original draft preparation: J. Paul Robinson, Grzegorz B. Gmyrek, and Bartek Rajwa. Writing – review and editing: J. Paul Robinson, Grzegorz B. Gmyrek, and Bartek Rajwa.

## Funding

The authors have nothing to report.

## Conflicts of Interest

J. Paul Robinson is a founder of Miftek Corporation.
